# DNMT1-mediated methylation inhibits microRNA-214-3p and promotes hair follicle stem cell differentiate into adipogenic lineages

**DOI:** 10.1186/s13287-020-01864-8

**Published:** 2020-10-19

**Authors:** Fangcao Jin, Min Li, Xuyang Li, Yunpeng Zheng, Kun Zhang, Xiaojun Liu, Bingjie Cai, Guangwen Yin

**Affiliations:** 1 Department of Dermatology, The First Affiliated Hospital of Zhengzhou University, No. 1, Jianshe East Road, Zhengzhou, 450052 Henan Province People’s Republic of China; 2 Department of Dermatology, Henan Provincial People’s Hospital, Zhengzhou, 450003 People’s Republic of China; 3 School of Life Sciences, Zhengzhou University, Zhengzhou, 450001 People’s Republic of China; 4 Henan Province Medical Instrument Testing Institute, Zhengzhou, 450018 People’s Republic of China

**Keywords:** Hair follicle stem cells, DNA methyltransferase 1, microRNA-214-3p, Mitogen-activated protein kinase 1, ERK1/2, Adipogenesis

## Abstract

**Background:**

Dysfunction of the DNA methylation was associated with stem cell reprogramming. Moreover, DNA methyltransferase 1 (DNMT1) deficiency was involved in the differentiation of hair follicle stem cell (HFSc), but the molecular mechanisms remain unknown.

**Methods:**

HFSc from human scalp tissues were isolated and cultured. The oil red O staining was used to observe the adipogenesis. The interaction relationship between microRNA (miR)-214-3p and mitogen-activated protein kinase 1 (MAPK1) was accessed by dual-luciferase reporter gene assay. The methylation level of miR-214-3p promoter was detected by methylation-specific PCR and the enrichment of DNMT1 in miR-214-3p promoter by chromatin immunoprecipitation assay. A mouse model of trauma was established to observe the skin regeneration at 0, 6, and 14 days.

**Results:**

Expression of DNMT1 and MAPK1 was increased in the HFSc, while the expression of miR-214-3p was reduced. Moreover, DNMT1 inhibited the expression of miR-214-3p by promoting the promoter methylation of miR-214-3p. Overexpression of DNMT1 could reduce the expression of miR-214-3p, but increase the expression of MAPK1 and the extent of extracellular signal regulated kinase (ERK)1/2 phosphorylation, leading to enhanced adipogenic differentiation. Importantly, DNMT1 promoted skin regeneration in vivo. Conversely, overexpression of miR-214-3p could reverse the effects of DNMT1 on adipogenesis of HFSc.

**Conclusion:**

DNMT1 promotes adipogenesis of HFSc by mediating miR-214-3p/MAPK1/p-ERK1/2 axis. This study may provide novel biomarkers for the potential application in stem cell therapy.

## Background

In the early stage of skin trauma, the basal cells at the edge of the wound begin to proliferate and form a single layer of epithelium, which covers the surface of the granulation tissues of the wound and then differentiate into squamous epithelium [1, 2]. Differentiation of epithelial cells is the key process of epidermal wound repair. Many complicated diseases, such as venous insufficiency (venous ulcer) and diabetes (diabetic foot ulcer), can slow down the differentiation of epithelial cells [3]. Moreover, skin differentiation and regeneration has been reported to rely on the transition from myofibroblasts into adipocytes [4]. In addition, diverse stem cells of the hair follicle (HF) and interfollicular epidermis have been found to accumulate at the site of skin injury [5]. Accumulating evidence suggests that epigenetic regulation of gene expression affects a variety of stem cell phenotypes [6]. The dynamic epithelial-mesenchymal crosstalk characteristic of hair follicle stem cells (HFSc) from embryonic development to adulthood can be found during normal hair follicle growth and cyclic regeneration [7]. Interestingly, emerging studies have revealed important functions of DNA methylation in skin biology such as it can regulate HFSc differentiation [8, 9]. DNA methylation is an epigenetic modification to change the epigenome that was mediated by DNA methyltransferase (DNMT). DNMT1 plays a central role in DNA methylation and can inhibit several microRNAs (miRNAs) and accumulates in the promoter regions of miRNAs [10]. However, the role of DNMT1 in wound healing and skin regeneration remains poorly understood.

A recent study reported that about 70 miRNAs play a role in controlling the development and differentiation of skin stem cells [11]. Some studies found that over 200 miRNAs were aberrantly expressed during the regeneration process of HFSc in the skin of mice [12]. Induction of dermal deletion in *Dicer* or *Drosha* in the skin of mice after birth also demonstrated the important role of miRNAs in maintaining the normal process of growth cycle and differentiation of HFSc [13]. More importantly, emerging evidence now supports the idea that DNA methylation is crucially involved in the dysregulation of miRNAs in many diseases, such as cancers, metabolic disorders, and atherosclerosis [14–16]. Of note, short interfering RNA (siRNA)-mediated knockdown of DNMT1 has been indicated to restore the expression of miR-214 in testicular germ cell tumor [17]. More importantly, miR-214 is implicated in self-renewal of skin tissues and found to be significantly downregulated in proliferation and differentiation of HFSc into transit-amplifying cells [18]. In the present study, we aim to detect the relationship between DNMT1 and miR-214-3p in the process of HFSc differentiation into adipogenic lineages.

## Materials and methods

### Ethics statement

This study was approved by the ethics committee of the First Affiliated Hospital of Zhengzhou University. The informed consent of the patients was obtained, and this study was performed following the principles recommended by the *Declaration of Helsinki*. All experimental procedures involving animals were performed in accordance with animal protocols approved by the Institutional Animal Use and Care Committee.

### HFSc isolation

Scalp tissues were obtained from 9 patients (6 males and 3 females) with a scalp laceration and contusion at the First Affiliated Hospital of Zhengzhou University. HFScs were isolated as described previously [18] with minor changes to the protocols. The scalp tissues were rinsed with penicillin-containing Hank’s solution for 3 times and penicillin-free Hank’s solution for 2 times. The scalp tissues were cut into 2 mm × 2 mm size and detached with 0.48 U/mL neutral protease overnight at 4 °C. The intact hair follicles were gently extracted from hair follicles with surgical tweezers. Then, 0.05% trypsin and 0.02% ethylenediaminetetraacetic acid mixture were added into the hair follicle tissues for detachment at 37 °C for 30 min, which was terminated by adding fetal bovine serum (FBS, C0265, Beyotime, Shanghai, China). The sample was then filtered with 100 mesh steel mesh and collected into a centrifuge tube. The supernatant was removed through centrifugation at 1000 rpm for 5 min. Dulbecco’s modified Eagle medium (DMEM) F12 (3:1), insulin (5 mg/L), transferrin (5 mg/L), hydrocortisone (0.4 mg/L), epidermal growth factor (EGF) (20 μg/L), amphotericin B (2.5 mg/L), penicillin (1051 U/ L), streptomycin (100 mg/L), and 20% FBS were added into centrifugal. The cells were cultured with 5% CO_2_ at 37 °C under saturated humidity. Finally, flow cytometer was used to detect the surface markers of HFSc including CK14, CD200, Integrin α6, and p63 to identify the successful isolation.

### Differentiation and transfection of HFSc

HFScs were differentiated into adipocytes according to previously described methods [19]. In brief, primary HFScs were cultured in DMEM containing 10% FBS and 1% penicillin/streptomycin (growth medium) in a 5% CO_2_ humidified atmosphere at 37 °C. Forty-eight hours after confluence, differentiation was induced with DMEM supplemented with 10% FBS, 1 μM dexamethasone, 10 μg/mL insulin, 0.2 mmol/L indomethacin, 0.1 mmol/L ascorbic acid, and 0.5 mM 3-isobutyl-1-methylxanthine (MDI medium). Two days later, the medium was replaced with DMEM (10% FBS) and was renewed every 2 days.

To construct lentiviral vectors overexpressing DNMT1 and MAPK1 and miR-214-3p, the human DNMT1 and MAPK1 and miR-214-3p DNA fragment was amplified by polymerase chain reaction (PCR) from human SGC7901 cell genomic DNA. The PCR-amplified fragments were inserted into a lentiviral vector pLV-EF1α-MCSIRES-Puro (pLV-ctrl) to generate pLV-DNMT1 and pLV-MAPK1 and pLV-miR-214-3p. Viral vector pLV-DNMT1 and pLV-MAPK1 and pLV-miR-214-3p and pLV-ctrl were transfected into HEK293T cells. Media containing lentiviruses (pLV-DNMT1 and pLV-MAPK1 and pLV-miR-214-3p and pLV-ctrl) were collected every 24 h for three times, and the lentiviruses were purified by ultra-speed centrifugation. Full-length cDNA encoding human DNMT1 and MAPK1 and miR-214-3p were amplified by PCR, and the PCR product was sub-cloned into pBOBI and pCMV-HA vectors to obtain DNMT1 and MAPK1 and miR-214-3p overexpressing plasmids.

For cell transient transfection, the HFScs were seeded and grown in 6-well plates overnight. The siRNA was used for inhibiting endogenous RNA expression [20]. The HFScs were transfected with negative control (NC) siRNA oligonucleotides, positive control siRNA oligonucleotides, siRNA against DNMT1 and miR-214-3p, using by Lipofectamine 2000 reagent (Invitrogen, Carlsbad, CA, USA) according to the manufacturer’s protocol. In addition, the HFScs were transfected with DNMT1 and MAPK1 and miR-214-3p overexpressing plasmids. The PD98059 (19-143, Sigma, St. Louis, MO, USA), a MAPK1 inhibitor, was also adopted to treat HFScs. Subsequent experiments were carried out 48 h after transfection.

### Reverse transcription-quantitative polymerase chain reaction (RT-qPCR)

TRIzol (Invitrogen, Carlsbad, CA, USA) was used to extract total RNA from cells, and the total RNA concentration and purity were detected by nanodrop2000 microultraviolet spectrophotometer (1011U, Nanodrop, USA). The RNA was reversely transcribed to complementary (cDNA) according to the instructions of TaqMan MicroRNA Assays Reverse Transcription primer (4427975, Applied Biosystems, USA), and the primer of miR-214-3p was designed and synthesized by TaKaRa (Table 1) with U6 as a control. For miRNAs, qPCR was performed with the stem-loop primers as reported previously [21].

**Table 1:** Primer sequences for RT-qPCR

	Primer sequences
miR-214-3p	F: 5′-ACACTCCAGCTGGGACAG-3′
	R: 5′-CTCGCTTCG GCAGCACA-3′
U6	F: 5′-CTCGCTTCGGCAGCACA-3′
	R: 5′-AACGCTTCACGAATTTGCGT-3′

*RT-qPCR* reverse transcription quantitative polymerase chain reaction, *miR* microRNA, *F* forward, *R* reverse

### Western blot analysis

Total protein was extracted from radio immunoprecipitation assay lysis buffer containing phenylmethanesulfonyl fluoride (P0013C, Beyotime Biotechnology, Shanghai, China) on ice for 30 min, centrifuged at 8000×*g* for 10 min at 4 °C. The 50 μg protein was dissolved in 2 × sodium dodecyl sulfate (SDS) loading buffer and boiled at 100 °C for 5 min. Proteins were separated by electrophoresis on 8 to 12% SDS-polyacrylamide gels and transferred moistly to polyvinylidene difluoride membranes. Membranes were blocked by 5% nonfat dry milk in phosphate-buffered saline and incubated with antibodies for DNMT1 (1:1000, ab188453, Abcam, Cambridge, UK), MAPK1 (1:200, R&D, MAB1230-SP), ERK1/2 (1:10,000, ab184699, Abcam), phosphorylated (p)-ERK1/2 (1:1000, ab214362, Abcam), peroxisome proliferators-activated receptor-γ2 (PPAR-γ2) (1:500, ab23673, Abcam), perilipin (1:1500, ab3526, Abcam), Adipoq (1:1000, ab62551, Abcam), aP2 (1:1000, ab218107, Abcam), and GAPDH (1:2500, ab9485, Abcam) overnight at 4 °C. The membrane was incubated with horseradish peroxidase-labeled goat anti-rabbit immunoglobulin G (IgG) (1:2000, ab97051, Abcam) for 1 h. The enhanced chemiluminescence fluorescence detection kit (BB-3501, Amersham, UK) was added on the membrane, and then, Bio-Rad image analysis system (Bio-Rad, USA) with Quantity One v4.6.2 analysis software was employed for analysis, and GAPDH was used as an internal control.

### Oil red O staining

The HFScs were cultured in DMEM/F12 and collected on the 7th day and 14th day, respectively. The cells were fixed with 10% formalin, washed with 60% isopropanol, and stained with oil red O working fluid. Being fixed by glycerine gelatin, cells were observed under a microscope (Olympus optics, Tokyo, Japan). The number of positive cells stained with oil red O was counted under the microscope.

### Dual-luciferase reporter gene assay

MAPK1 was identified as a miR-214-3p target in TargetScan7.1 (http://www.targetscan.org/vert_71/). Human HEK293T cells were cultured in DMEM containing 10% FBS. The cDNA fragment of MAPK1 3′-untranslated region (3′-UTR), MAPK1-wild type (Wt) containing the miR-214-3p binding site was inserted into the pmiRGLO vector. The cDNA fragment of MAPK1 3′-UTR, MAPK1-mutant type (Mut) was synthesized by point mutation and inserted into pmiRGLO vector. The inserted sequence was verified to be correct by sequencing performed by RiboBio Co., Ltd. (Shanghai, China). The recombinant vector pmiRGLO-MAPK1-Wt or pmiRGLO-MAPK1-Mut was co-transfected with a miR-214-3p mimic (miR-214-3p overexpression sequence) or an NC mimic (negative control sequence) into HEK293T cells by liposome transfection, and the cells were incubated and cultured for 48 h before being collected and lysed. Then, 100 μL lysate supernatant was taken and 100 μL Renilla luciferase assay solution was added to detect the Renilla luciferase activity. In addition, 100 μL lysate supernatant and 100 μL firefly luciferase were added to detect the firefly luciferase activity. After 48 h, the cells were collected, and the luciferase and Renilla luciferase activities were determined using the Dual-Luciferase Reporter Assay System (Promega, Madison, WI, USA). Firefly luciferase activity was normalized to Renilla luciferase activity. The SpectraMax M5 (Molecular devices instruments Co., Ltd., Shanghai, China, Origin: USA) at an interval of 2 s and a determination time design of 10 s were used to detect the activity of Renilla luciferase and firefly luciferase, respectively.

### Real-time quantitative methylation-specific PCR

The methylation of gene promoter was detected by methylation-specific PCR (MSP). Genomic DNA was extracted by Genomic DNA extraction kit (Tiangen Biochemical technology Co., Ltd., Beijing, China) according to the instructions. The DNA concentration and purity were determined by ultraviolet spectrophotometry. DNA was treated with sodium sulfite using the EZ DNA Methylation Kit (Zymo Research, USA), and the reaction column was used for desulfurization and purification. The purified DNA could be used for subsequent PCR. The methylation and non-methylation primers (Table 2) were designed for the miR-214-3p promoter by CpG island enrichment area. The reaction products were subjected to agarose gel electrophoresis, gel electrophoresis imaging, and image analysis system. If the CpG island in the promoter region is completely methylated, only the methylated primer can amplify the target band. If there is no methylation, only non-methylated primers can amplify the target band. If partial methylation occurs, the target bands can be amplified from both primers. Partial methylation is classified as methylation. Serial dilutions of plasmid DNA were used as standards for quantification [22].

**Table 2:** Primer sequences for MS-PCR

	Primer sequences
miR-214-3p-M	F: 5′-TTGTTTGGTATTCGGGTTTTC-3′
	R: 5′-AAAAAATAAAAAAAATTCTTTCGTT-3′
miR-214-3p-U	F: 5′-TTTTTGTTTGGTATTTGGGTTTTT-3′
	R: 5′-AAAAAATAAAAAAAATTCTTTCATT-3′

*MS-PCR* methylation-specific polymerase chain reaction, *miR* microRNA, *F* forward, *R* reverse, *M* methylated, *U* unmethylated

### Chromatin immunoprecipitation (ChIP)

Differentiated and cultured HFScs were taken, the cell fusion degree reached 70–80%, and 1% formaldehyde was added to fix cells at room temperature for 10 min to make the DNA and protein fixed and cross-linked. Then, the protein was broken randomly by ultrasonic treatment for 10 s, at an interval of 10 s, 15 cycles in total to make fragments of appropriate size. The supernatant was collected by centrifugation at 13,000 rpm and divided into two tubes, which was added with antibody for NC and rabbit anti-IgG (1:100, ab172730, Abcam) and mouse anti-DNMT1 (1:100, ab13537, Abcam) at 4 °C overnight, respectively. The endogenous DNA-Protein complex was precipitated by Protein Agarose/Sepharose, and the non-specific complex was washed, the cross-linking was performed overnight at 65 °C, and the DNA fragments were extracted and purified by phenol and chloroform to detect the binding of miR-214-3p promoter fragment to DNMT1.

### Mouse model of skin trauma

A total of 24 mice (weight 20 ± 2 g) were purchased from Hunan SJA Laboratory Animal Co., Ltd. (Changsha, Hunan, China). The mice were anesthetized by intraperitoneal injection with 3% barbiturate. Two wounds were created at 1.0 cm to both sides of the back-spinal column to form a circular skin incision without touching the muscles. After the wound was formed, it was not bandaged and treated with medicine. The mice were kept separately in a sterile laboratory and sterilized every day. Lentivirus vectors (5 × 10^8^ pfu/100 L) were introduced into the wound surface of mice beside the wound surface. The mice were randomly divided into oe-NC + sh-NC group (*n* = 8), oe-DNMT1 + sh-NC group (*n* = 8), and oe-DNMT1 + sh-MAPK1 group (*n* = 8). The overexpression lentivirus vectors were used by LV5-GFP, and silencing lentivirus vectors were used based on pSIH1-H1-copGFP. The wound area of each group was photographed and recorded on days 0, 6, 10, and 14, respectively. The mice were sacrificed on day 18, and skin tissues were extracted from the wound. The tissue sections were embedded by paraffin or used to extract proteins for detection.

### Immunofluorescence staining

Mouse skin tissues were fixed with 4% paraformaldehyde for overnight. Then, the tissues were washed, sealed with normal saline of 0.01 M phosphate buffer for three times, and then sealed with 10% goat serum (C0265, Beyotime, Shanghai, China) at room temperature for 30 min. After that, transforming growth factor-β1 (TGF-β1) (1:200, ab92486, Abcam), vascular endothelial growth factor (VEGF) (1:200, ab2350, Abcam), and platelet-derived growth factor (PDGF-BB) (1:200, ab9704, Abcam) were incubated with tissues at 4 °C for overnight. The secondary antibody and 4′,6-diamidino-2-phenylindole were incubated at room temperature in the dark for 1 h, and glycerol was fixed. The confocal laser scanning microscope was used for analysis (LSM, FV1000; Olympus Corp., Tokyo, Japan).

### Statistical analysis

All statistical tests were performed using SPSS 21.0 (IBM SPSS Statistics, Armonk, NY, USA). The data were presented as mean ± standard deviation. The data in the two groups were compared by unpaired Student’s *t* test, and the data in multiple groups were compared by one-way analysis of variance (ANOVA) and Tukey’s post-test. *p* < 0.05 was considered statistically significant.

## Results

### HFScs are successfully isolated

**Fig. 1:**
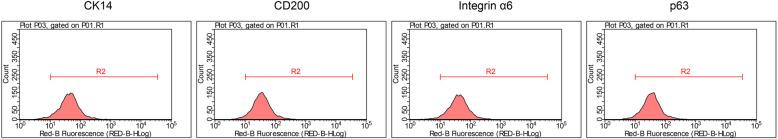
Flow cytometric analysis for surface markers of HFScs

The markers for HFSc at passage 3, CK14, CD200, Integrin α6, and p63 were tested, to confirm the isolation of HFSc. There was an increased expression of CK14, CD200, Integrin α6, and p63 in HFSc (Fig. 1), suggesting that HFScs were successfully isolated.

### DNMT1 promotes differentiation of HFSc into adipogenic lineages

**Fig. 2:**
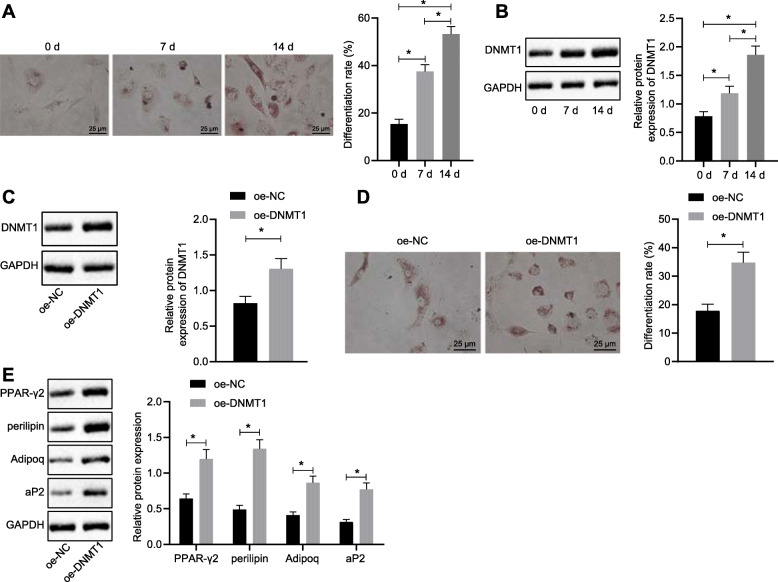
DNMT1 promotes adipogenic differentiation of HFSc. **a** Oil red O staining was used to detect the adipogenic differentiation of HFSc. **b** Western blot analysis detected the expression of DNMT1 normalized to GAPDH in HFSc cultured in differentiation medium for 0 days, 7 days, and 14 days. **c** Western blot analysis detected the expression level of DNMT1 by overexpression of DNMT1 normalized to GAPDH. **d** The degree of adipogenic differentiation of HFSc after overexpression of DNMT1 was detected by oil red O staining. **e** Western blot analysis was used to detect the expression level of adipose formation markers normalized to GAPDH. Data were expressed as mean ± standard deviation. **a**, **b** By one-way ANOVA and Tukey’s post hoc test. **c**–**e** By unpaired Student’s *t* test, *n* = 3 per group, * *p* < 0.05

In order to demonstrate the effect of DNMT1 in the differentiation of HFSc, the HFScs were induced differentiation into adipogenic lineages. According to oil red O staining, lipid droplets were detected in the cytoplasm on the 7th day after induction and progressively increased to the 14th day (Fig. 2a), suggesting that HFScs have successfully undergone differentiation of adipogenic lineages. The expression of DNMT1 was increased on the 7th day and peaked on the 14th day by successful induction (Fig. 2b). After transfection with overexpressing DNMT1 vectors, the expression of DNMT1 was increased (Fig. 2c), and the proportion of differentiated cells was significantly upregulated as revealed by oil red O staining (Fig. 2d). Moreover, the expression of adipose formation markers, PPAR-γ2, perilipin, Adipoq, and aP2, were significantly increased by overexpressing of DNMT1 (Fig. 2e). These data indicated that DNMT1 promotes HFSc differentiate into adipogenic lineages.

### DNMT1 promotes differentiation of HFSc into adipogenic lineages by downregulating miR-214-3p expression

**Fig. 3:**
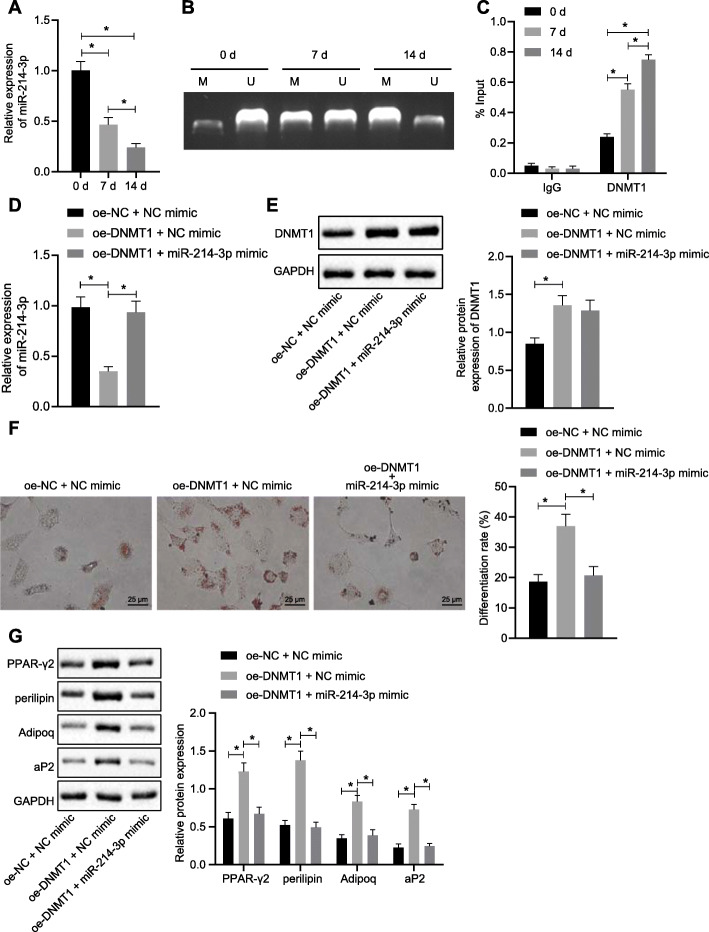
DNMT1 promotes HFSc differentiate into adipogenic lineages by inhibition of miR-214-3p. **a** The expression level of miR-214-3p was detected by RT-qPCR after the adipogenic differentiation of HFSc. **b** MSP detected the methylation level of miR-214-3p promoter after the adipogenic differentiation of HFSc. **c** ChIP detected the enrichment of DNMT1 on the miR-214-3p promoter after the HFScs were cultured in differentiation medium for 0 days, 7 days, and 14 days. **d** The expression level of miR-214-3p in each group was detected by RT-qPCR. **e** Western blot analysis was used to detect the expression level of DNMT1 normalized to GAPDH in each group. **f** The degree of adipogenic differentiation of HFSc was detected by oil red O staining. **g** Western blot analysis was used to detect the expression level of adipose formation markers normalized to GAPDH. The data were expressed as mean ± standard deviation. *n* = 3 per group. *p < 0.05

Subsequently, we found the expression of miR-214-3p was reduced on the 7th day and lowest on the 14th day over differentiation (Fig. 3a). The MSP showed that the methylation level of miR-214-3p promoter was significantly increased on the 7th day and the 14th day by differentiation (Fig. 3b). We further investigated whether DNMT1 can directly influence differentiation process of HFSc by methylation effects in the promoter region of the miR-214-3p. We found that the enrichment of DNMT1 on miR-214-3p promoter was significantly increased on the 7th day and the 14th day by differentiation (Fig. 3c).

To detect the role of miR-214-3p in the process of DNMT1 promoting HFSc differentiation, we test the mRNA and protein level of DNMT1 in different HFSc groups. We found that overexpression of DNMT1 could significantly reduce the level of miR-214-3p, which could be reversed by administration of miR-214-3p mimic (Fig. 3d, e). The oil red O staining showed the proportion of differentiated cells was significantly increased in the oe-DNMT1 + NC mimic group than that in the oe-NC + NC mimic group, which was restored in the oe-DNMT1 + miR-214-3p mimic group (Fig. 3f). Moreover, the expression level adipose formation markers, PPAR-γ2, perilipin, Adipoq, and aP2, were higher in the oe-DNMT1 + NC mimic group than that in the oe-NC + NC mimic group, and which was restored in the oe-DNMT1 + miR-214-3p mimic group (Fig. 3g).

### miR-214-3p inhibits adipogenic differentiation of HFSc by inhibiting MAPK1

**Fig. 4:**
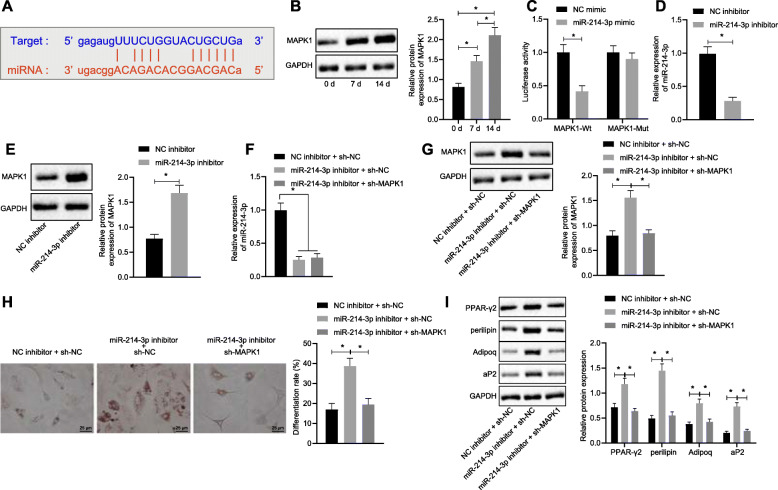
miR-214-3p inhibits adipogenic differentiation of HFSc by downregulating MAPK1. **a** The binding sites between miR-214-3p and MAPK1 mRNA in 3′-UTR were predicted. **b** Western blot analysis was used to detect the expression of MAPK1 normalized to GAPDH in HFSc after adipogenic differentiation. **c** Dual-luciferase reporting experiment verified the targeting relationship between miR-214-3p and MAPK1. **d** Transfection efficiency of miR-214-3p after downregulating of miR-214-3p by RT-qPCR. **e** MAPK1 expression level normalized to GAPDH after inhibition of miR-214-3p was detected by Western blot analysis. **f** The expression levels of miR-214-3p in each group were detected by RT-qPCR. **g** Western blot analysis was used to detect the expression level of MAPK1 normalized to GAPDH in each group. **h** The degree of adipogenic differentiation of HFSc was detected by oil red O staining. **i** Western blot analysis was used to detect the expression level of adipose formation markers normalized to GAPDH. The data were expressed as mean ± standard deviation. *n* = 3 per group. *p < 0.05

To detect the regulation mechanism of miR-214-3p, we predicted through the Starbase2 (http://starbase.sysu.edu.cn/starbase2/) and found MAPK1 might be the downstream regulatory gene of miR-214-3p (Fig. 4a). More importantly, Cai et al. showed that MAPK1 promotes HFSc proliferation, wound contraction, and epidermal regeneration in mouse model [23]. Here, we found the expression of MAPK1 was significantly increased in HFSc by differentiation on the 7th day and the 14th day (Fig. 4b). To investigate the potential targeting of MAPK1 by miR-214-3p, a luciferase activity assay was designed. Expression of miR-214-3p in 293T and SGC7901 cells obviously inhibited the luciferase activity of the MAPK1-3′-UTR reporter. Additionally, our qPCR analyses indicated that, compared to the control transfected group, transfection of 293T and SGC7901 cells with miR-214-3p significantly increased miR-214-3p expression (Fig. 4c), suggesting that MAPK1 mRNA was subjected to post-transcriptional control of miR-214-3p by targeting the MAPK1-3′-UTR. We further transfection HFSc with miR-214-3p inhibitor to decrease the expression of miR-214-3p (Fig. 4d), but the expression of MAPK1 was significantly increased by miR-214-3p inhibitor (Fig. 4e). These data indicated that miR-214-3p can target to inhibit MAPK1.

To explore whether miR-214-3p affect adipogenic differentiation of HFSc by regulating MAPK1, the HFScs were incubated with inhibitor of miR-214-3p and MAPK1. We found the expression level of miR-214-3p was reduced by inhibition of miR-214-3p, while the gene expression level of MAPK1 was increased (Fig. 4f). Similar results were confirmed by Western blot analysis (Fig. 4g). The oil red O staining showed the proportion of differentiated cells was significantly increased in the miR-214-3p inhibitor + sh-NC group than that in the NC inhibitor + sh-NC group, while the proportion of differentiated cells was significantly reduced in the miR-214-3p inhibitor + sh-MAPK1 group (Fig. 4h). A similar tendency was observed on the detection of the expression levels of adipose formation marker protein including PPAR-γ2, perilipin, Adipoq, and aP2 (Fig. 4).

### miR-214-3p inhibits adipogenic differentiation of HFSc by reducing MAPK1-mediated expression of p-ERK1/2

**Fig. 5:**
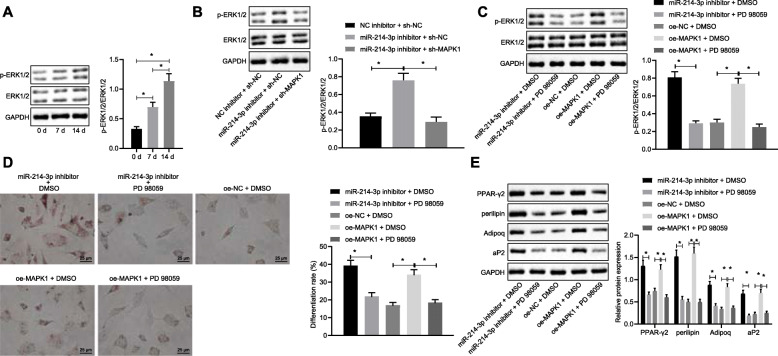
miR-214-3p inhibits adipogenic differentiation of HFSc by reducing MAPK1-mediated expression of p-ERK1/2. **a** Western blot analysis was used to detect the phosphorylation level of ERK1/2 normalized to GAPDH in HFSc after adipogenic differentiation of HFSc. **b** Western blot analysis detected the phosphorylation level of ERK1/2 normalized to GAPDH in each group. **c** Western blot analysis detected the phosphorylation level of ERK1/2 normalized to GAPDH in each group. **d** Oil red O staining was used to detect the degree of adipogenic differentiation of HFSc. **e** Western blot analysis was used to detect the expression level of adipose formation markers normalized to GAPDH. *n* = 3 per group. *p < 0.05

Previous studies showed that MAPK1 promotes the expression of p-ERK1/2 [24], while the EGF that promotes the extent of ERK1/2 phosphorylation [25] could induce the differentiation of HFSc, suggesting that p-ERK1/2 may play an important role in the differentiation process of HFSc. Here, we found the expression of phosphorylation of ERK1/2 was significantly increased in HFSc by differentiation on the 7th day and the 14th day (Fig. 5a). We also found the extent of ERK1/2 phosphorylation was significantly increased by miR-214-3p inhibitor, while it was reversed by miR-214-3p inhibitor and sh-MAPK1 (Fig. 5b) suggesting that miR-214-3p could promote the extent of ERK1/2 phosphorylation by regulating MAPK1.

To test whether miR-214-3p promotes MAPK1 by regulating p-ERK1/2, the PD 98059, an inhibitor of p-ERK1/2, was administrated in HFSc. Here, we found overexpression of MAPK1 significantly enhanced the expression level of p-ERK1/2, while PD 98059 reversed the effect (Fig. 5c). The oil red O staining showed the proportion of differentiated cells was significantly reduced in the miR-214-3p inhibitor + PD 98059 group, while it was significantly increased by overexpression of MAPK1. In addition, the effect of MAPK1 was restored by PD 98059 (Fig. 5d). Moreover, the similar results of adipose formation marker protein including PPAR-γ2, perilipin, Adipoq, and aP2 were observed by Western blot analysis (Fig. 5e). Taken together, these data indicated that miR-214-3p inhibits adipogenic differentiation of HFSc by reducing MAPK1-mediated expression of p-ERK1/2.

### DNMT1 activates the MAPK1/p-ERK1/2 axis by downregulating miR-214-3p, thereby promoting wound healing and skin regeneration in mice

**Fig. 6:**
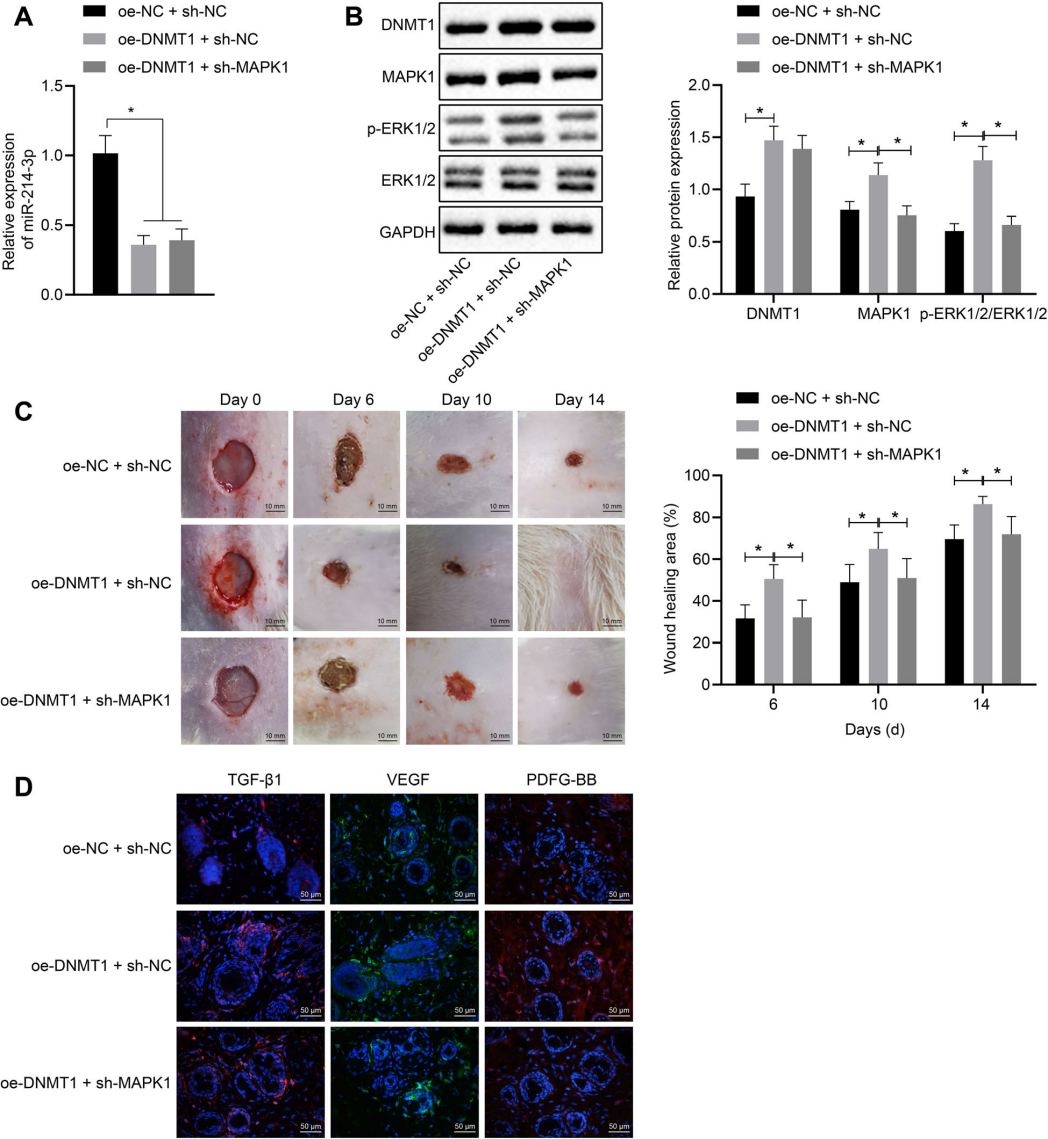
DNMT1 activates the MAPK1/p-ERK1/2 axis by downregulating miR-214-3p, thereby promoting wound healing and skin regeneration in mice. **a** The expression levels of miR-214-3p in each group were detected by RT-qPCR. **b** Western blot analysis detected the phosphorylation levels of DNMT1 and ERK1/2 normalized to GAPDH in each group. **c** Epidermal regeneration and quantitative analysis of epidermal tissues in each group. **d** Immunofluorescence staining was used to detect wound healing growth factors in wound tissues of each group. *n* = 8 per group. *p < 0.05

The mouse model of skin trauma was established to test the effect of DNMT1 on the adipogenic differentiation of HFSc. The expression of DNMT1, MAPK1, and p-ERK1/2 was increased and miR-214-3p expression reduced in the oe-DNMT1 + sh-NC group, while the expression levels of MAPK1 and p-ERK1/2 gene were reduced in the oe-DNMT1 + sh-MAPK1 when compared with the oe-DNMT1 + sh-NC group (Fig. 6a). The similar expression tendency of protein was found by Western blot analysis (Fig. 6b). Here, we found the epidermis of mice in each group showed different degrees of growth and recovery, and the skin regeneration peaked by overexpression of DNMT1 that reserved by sh-MAPK1 (Fig. 6c). Moreover, the wound healing growth factors including TGF-β1 and VEGF and PDGF-BB in all groups were detected by immunofluorescence with red color indicative of the expression of TGF-β1 or PDGF-BB, and with green color indicative of VEGF expression. Compared with the oe-NC + sh-NC group, the expression of TGF-β1 and VEGF and PDGF-BB were significantly increased in the oe-DNMT1 + sh-NC group, while it was restored in the oe-DNMT1 + sh-MAPK1 group (Fig. 6d). These data indicated that DNMT1 increased the MAPK1/p-ERK1/2 axis by downregulating miR-214-3p, thereby promoting wound healing and skin regeneration in mice.

## Discussion

DNA methylation has been elucidated as a critical molecular mechanism to be of importance to the skin wound healing process and nociceptive sensitization following skin incision [2, 26]. Additionally, DNMTs are known to participate in the self-renewal and senescence of human HFSc, which are recognized to be recruited to the skin injury site for further differentiation to repair injured epithelium [27]. In the present study, we found that DNMT1 gene was upregulated in adipogenic differentiation of HFSc. In addition, our results showed that miR-214-3p expression was reduced when its promoter was hypermethylated in adipogenic differentiation of HFSc. Moreover, treatment with MAPK1 could restore the proportion of differentiated cells in cultured adipogenic differentiation of HFSc by miR-214-3p. Altogether, hypermethylation of miR-214-3p promoter was regarded as a primary source for the loss of miR-214-3p expression in adipogenic differentiation of HFSc. As a result, the skin regeneration was enhanced by overexpression of DNMT1 in the mouse model of skin trauma. Thus, our findings may help to clarify the mechanism of adipogenic differentiation of HFSc and address the possible relationship between DNMT1 and miR-214-3p in the differentiation process of HFSc.

DNA methylation is an epigenetic modification method which is mediated by DNMT. DNMTs consist of three subtypes, including DNMT1, DNMT2, and DNMT3, which catalyze the methylation of cytosine bases and cause changes in apparent modification. DNMT1 is a kind of semi-methylated DNMT and can accumulate in the promoter region of many genes, leading to gene silencing. Previous study has found that DNMT1 gene deficiency can reduce the activation rate of stem cells in the aging process of mice, suggesting that DNMT1 is closely related to stem cell differentiation [28]. Moreover, the expression of DNMT1 is reduced in de-differentiation of HFSc [29], suggesting that DNMT1 may play an important role in the differentiation process of HFSc. In the present study, we found that inducing epidermal stem cell differentiation can significantly increase the expression of DNMT1, while overexpression of DNMT1 can significantly increase the proportion of epidermal stem cell differentiation, suggesting that DNMT1 can promote epidermal stem cell differentiation by inducing DNA methylation. Of note, somatic cell reprogramming of human HFSc has been found to be correlated with DNMT1 deficiency, involving the mediation of miR-302 [24]. Numerous studies have shown that DNA methylation and miRNA influence each other and maintain the stability of the body in a balanced relationship. For example, the promoters of miR-122, miR-129, and miR-191 are affected by DNA methylation and participate in the occurrence of a variety of tumors. Chen et al. found that inhibition of DNMT1 activity could upregulate the expression by inducing miR-214-3p DNA demethylation [17], and miR-214-3p was reduced in the differentiation process of HFSc [18]. Therefore, we speculated that DNMT1 may affect the differentiation of HFSc by inhibiting the expression of miR-214-3p. In the present study, we found that the expression of miR-214-3p was reduced in lipogenic HFSc, and the methylation level of miR-214-3p promoter was significantly increased, suggesting that the adipogenic differentiation of HFSc may be caused by miR-214-3p DNA methylation. In addition, we found that miR-214-3p could reverse the differentiation of HFSc induced by overexpression of DNMT1. In order to further clarify the molecular mechanism of the regulation of miR-214-3p by DNMT1, we used ChIP and dual-luciferase reporter gene detection to confirm the direct effect of DNMT1 and miR-214-3p.

Although it has been found that inhibition of miR-214-3p could induce HFSc differentiation, the specific molecular mechanism has not been elucidated. Through the prediction of the website, we found the downstream binding target MAPK1 of miR-214-3p. In the present study, we found that miR-214-3p can directly inhibit the expression of MAPK1, and the dual-luciferase reporter gene assay also confirmed the direct binding between miR-214-3p and MAPK1 gene. Cai et al. showed that MAPK1 could promote HFSc proliferation, wound contraction, and epidermal regeneration of mouse model [23]. Consistent with the previous study, we found that inhibition of miR-214-3p could induce HFSc differentiation, while inhibition of MAPK1 can reverse this effect, suggesting that MAPK1, as a downstream effector molecule of miR-214-3p, plays a role in promoting HFSc differentiation. ERK1/2 is a classical effector molecule downstream of MAPK1. Here, we found that miR-214-3p inhibits the adipogenic differentiation of HFSc by inhibiting the MAPK1-mediated upregulated p-ERK1/2. Finally, we constructed a wound healing mouse model and found that overexpression of DNMT1 could promote the skin regeneration, while inhibition of MAPK1 could reverse the above effects. More importantly, stem cell therapies have been widely used in various fields; for instance, HFSc can be employed as a treatment modality for hair loss [30]. Hair growth, another skin regeneration process in addition to skin wound healing, has been elaborated to be promoted by adipose-derived stem cells as well [31]. Furthermore, platelet-rich plasma (PRP), belonging to autologous growth factors, has been demonstrated to play an important role in hair growth [32]. Also, available evidence has confirmed the application of PRP in combination with hyaluronic acid to wound healing process of both soft and hard tissues [33], including bone tissue defects [34] and scar correction on the face [35], suggesting the need of further exploration on the clinical application value of HFSc.

In conclusion, the present study confirmed that DNMT1 can impair miR-214-3p-mediated inhibition of MAPK1 and promote HFSc differentiation by increasing the methylation of miR-214-3p promoter. In injured human epithelial cells, the gradual loss of DNMT1 protein or enzyme activity has been reported, indicating that the loss of DNMT1 in epithelial cells is partially responsible for the process of skin regeneration. Future work will identify more DNMT-mediated molecular targets in stem cells and further clarify the relationship between epidermal DNMT defects and human skin lesion repair.

## Data Availability

The authors confirm that the data supporting the findings of this study are available within the article.

## Abbreviations

DNMT1: DNA methyltransferase 1
HFSc: Hair follicle stem cell
miR: microRNA
MAPK1: Mitogen-activated protein kinase 1
EDTA: Ethylenediaminetetraacetic acid
DMEM: Dulbecco’s modified Eagle medium
FBS: Fetal bovine serum
siRNA: Short interfering RNA
